# Epigenomic biomarkers insights in PBMCs for prognostic assessment of ECMO-treated cardiogenic shock patients

**DOI:** 10.1186/s13148-024-01751-6

**Published:** 2024-10-03

**Authors:** Yi-Jing Hsiao, Su-Chien Chiang, Chih-Hsien Wang, Nai-Hsin Chi, Hsi-Yu Yu, Tsai-Hsia Hong, Hsuan-Yu Chen, Chien-Yu Lin, Shuenn-Wen Kuo, Kang-Yi Su, Wen-Je Ko, Li-Ming Hsu, Chih-An Lin, Chiou-Ling Cheng, Yan-Ming Chen, Yih-Sharng Chen, Sung-Liang Yu

**Affiliations:** 1 Department of Clinical Laboratory Sciences and Medical Biotechnology, College of Medicine, National Taiwan University, Taipei, Taiwan; 2 Institute of Chemistry, Academia Sinica, Taipei, Taiwan; 3 Center for Institutional Research and Data Analytics, National Yang Ming Chiao Tung University, Hsinchu, Taiwan; 4 Department of Surgery, National Taiwan University Hospital, Taipei, Taiwan; 5 Centers of Genomic and Precision Medicine, National Taiwan University, Taipei, Taiwan; 6 Institute of Statistical Science, Academia Sinica, Taipei, Taiwan; 7 Department of Laboratory Medicine, National Taiwan University Hospital, Taipei, Taiwan; 8 Department of Pathology and Graduate Institute of Pathology, College of Medicine,, National Taiwan University, Taipei, Taiwan; 9 Graduate School of Advanced Technology, , National Taiwan University, Taipei, Taiwan; 10 Institute of Medical Device and Imaging, College of Medicine, National Taiwan University, Taipei, Taiwan

**Keywords:** ECMO, Prognostic biomarker, Epigenome

## Abstract

**Objective:**

As the global use of extracorporeal membrane oxygenation (ECMO) treatment increases, survival rates have not correspondingly improved, emphasizing the need for refined patient selection to optimize resource allocation. Currently, prognostic markers at the molecular level are limited.

**Methods:**

Thirty-four cardiogenic shock (CS) patients were prospectively enrolled, and peripheral blood mononuclear cells (PBMCs) were collected at the initiation of ECMO (t0), two-hour post-installation (t2), and upon removal of ECMO (tr). The PBMCs were analyzed by comprehensive epigenomic assays. Using the Wilcoxon signed-rank test and least absolute shrinkage and selection operator (LASSO) regression, 485,577 DNA methylation features were analyzed and selected from the t0 and tr datasets. A random forest classifier was developed using the t0 dataset and evaluated on the t2 dataset. Two models based on DNA methylation features were constructed and assessed using receiver operating characteristic (ROC) curves and Kaplan–Meier survival analyses.

**Results:**

The ten-feature and four-feature models for predicting in-hospital mortality attained area under the curve (AUC) values of 0.78 and 0.72, respectively, with LASSO alpha values of 0.2 and 0.25. In contrast, clinical evaluation systems, including ICU scoring systems and the survival after venoarterial ECMO (SAVE) score, did not achieve statistical significance. Moreover, our models showed significant associations with in-hospital survival (*p* < 0.05, log-rank test).

**Conclusions:**

This study identifies DNA methylation features in PBMCs as potent prognostic markers for ECMO-treated CS patients. Demonstrating significant predictive accuracy for in-hospital mortality, these markers offer a substantial advancement in patient stratification and might improve treatment outcomes.

**Supplementary Information:**

The online version contains supplementary material available at 10.1186/s13148-024-01751-6.

## Introduction

Cardiogenic shock (CS) is characterized by acute hypoperfusion resulting from reduced cardiac output, commonly triggered by acute myocardial infarction (AMI), fulminant myocarditis, or dilated cardiomyopathy (DCMP) [1]. Venoarterial extracorporeal membrane oxygenation (VA-ECMO) utilizes peripheral vascular access, employing a pump to divert venous blood to a membrane oxygenator and then perfuse it back into the peripheral arteries, thereby providing partial cardiopulmonary bypass to support patients with cardiogenic shock due to myocardial infarction, cardiomyopathy, and other conditions, significantly enhancing survival probability [2]. Patients undergoing extracorporeal cardiopulmonary resuscitation (ECPR) have demonstrated a roughly 20% increase in survival rates at hospital discharge compared to those receiving conventional cardiopulmonary resuscitation (CPR) [3]. Despite these benefits, ECMO treatment is limited by potential neurological impairments and high mortality rates; survival rates for CS patients with ECPR remain low [4, 5]. Meta-analyses suggest that in-hospital survival for CS patients is generally between 30 and 45% [6–8]. Therefore, accurately identifying patients who are likely to respond to ECMO is crucial for enhancing outcomes and minimizing unnecessary treatments.

Establishing prognostic biomarkers at the early phase under ECMO support is an emerging urgency to help clinicians accurately making medical decisions and conducting management. The currently used important prognostic indicators for patient survival include age, ECMO supporting time, renal failure, obesity, and lactate, but the correlations between these parameters and survival are still controversial across various studies [7, 8]. For refractory cardiogenic shock patients receiving ECMO, the survival after venoarterial-ECMO (SAVE) scoring system is an available tool to predict survival [6]. The parameters include acute cardiogenic shock diagnosis, pre-ECMO organ failure, blood pressure, pulse pressure, etc. Predicting survival for cardiogenic shock (CS) patients receiving ECMO based on their physiological and clinical characteristics is established among previous studies, yet these parameters are not without limitations [6, 9, 10]. One significant challenge is the retrospective scoring of variables collected at the time of ECMO installation, which may not align with real-time decision-making processes [6]. It is essential to identify biomarkers or sets of variables that can accurately classify a patient’s responsiveness to ECMO support. In our previous research, IL-10 was identified as a promising marker potentially predictive of outcomes at the time of ECMO initiation for CS patients [11]. Although the area under the curve (AUC) for IL-10 exceeded 0.9, its basal levels varied significantly between patients with acute myocardial infarction (AMI) and those with dilated cardiomyopathy (DCMP) [11].

To address the complexity inherent in varying diseases, a generalized additive and linear model was developed. This model integrates dynamic changes in lymphocytes and interleukins measured in the early stages of ECMO installation [12]. However, the challenge of collecting and monitoring multiple variables at various time points during ECMO support remains a significant concern. Preoperative gene expression profiles in PBMCs have shown an average prediction accuracy of 93% for early improvement in organ function among advanced heart failure patients receiving mechanical circulatory support [13]. To date, only a limited number of studies have successfully demonstrated the identification of viable biomarkers using a genomic approach.

DNA methylation, which reflects genetic, environmental influences and age, holds promise as a biomarker in all-cause mortality, cancers, and various complex disease, including cardiovascular disease (CVD) [14, 15]. Previous studies demonstrated the potential of epigenetic biomarker in clinical applications. The methylation biomarkers derived from circulating cell-free DNA and monocyte-related profiles could differentiate between types of acute coronary syndrome and associate with CVD risk, respectively [16, 17]. The accumulating evidence implies the potential of epigenetic profiles in the development of prognostic markers for CS patients receiving ECMO.

Herein, our objective is to develop a prognostic model for cardiogenic shock (CS) patients to predict outcomes following ECMO resuscitation. In this study, comprehensive epigenomic analysis was conducted on PBMCs collected at various time points: initial (0 h), 2-h post-installation, and at the removal of ECMO. We developed a machine learning model using selected DNA methylation features to enhance prognostic significance.

## Methods

### Study population and sample preparation

This study was approved by the Research Ethics Committee of National Taiwan University Hospital with the approval reference number 200911043R. Trial registration: ClinicalTrials.gov, NCT01089036. Registered March 17, 2010—Prospectively registered, https://clinicaltrials.gov/study/NCT01089036. The inclusion criteria encompassed cardiogenic shock (CS) patients aged ≥ 18 years requiring ECMO support, while exclusion criteria included preexisting multiple organ failure (MOF), sepsis, severe brain insult prior to ECMO support, and lack of signed consent for study enrollment. Patients experiencing circulatory collapse or requiring inotrope support exceeding 40 inotropic equivalents were considered for ECMO therapy. The ECMO system was established using a femoral venoarterial route (Medtronic Inc., Minneapolis, MN) [3]. A total of 34 patients received ECMO treatment between July 21, 2010, and May 14, 2012. Prior studies suggest that the average duration of ECMO support is approximately 7 days, which we use as the primary outcome [18, 19]. Therefore, the primary outcome is set as one of our enrollment criteria for the epigenomic assay to ensure an even number of cases are enrolled.

In this study, 17 patients who survived more than 7-day post-ECMO installation were classified as early success, while those who did not survive or succumbed to MOF within 7 days were classified as failure. After continuous follow-up, the endpoint outcome was measured based on mortality. Patients who died during hospitalization were categorized as non-survivors (*n* = 22), whereas those discharged alive were classified as survivors (*n* = 12). Preexisting medical histories, comorbidities, demographic parameters, and risk scoring systems were collected and evaluated.

Blood was collected from all 34 patients at 0 h and 2 h after ECMO installation, and from 14 patients who successfully concluded ECMO support. Blood samples were collected using BD Vacutainer® Brand BD CPT™ Mononuclear Cell Preparation Tubes (BD Diagnostics, La Jolla, CA) containing sodium heparin, a separation gel, and a density gradient fluid for isolating mononuclear cells. Isolated PBMCs were stored in liquid nitrogen until analysis. DNA were extracted from each sample using the AllPrep DNA Micro Kit (Qiagen, Hilden, Germany) for DNA methylation analyses.

### Genome-wide DNA methylation assays

Genome-wide DNA methylation of PBMCs was assessed using the Illumina HumanMethylation450 BeadChip, which includes 485,577 methylation probes offering single-nucleotide resolution, following the manufacturer instructions (Illumina, San Diego, CA). It covers approximately 99% of RefSeq genes, enabling comprehensive and detailed epigenetic profiling across the entire human genome. Data were subsequently uploaded to the GEO database (GSE182609).

### Statistical analysis and model selection

Initial filtering of methylation probes was based on the Human Methylation450 BeadChip annotation file, with only probes annotated in the UCSC_RefGene being further analyzed. The Wilcoxon Signed-Rank Test assessed methylation level differences pre- and post-ECMO support. For classified model development, we utilized the least absolute shrinkage and selection operator (LASSO) regression for feature selection, leveraging its regularization properties to mitigate overfitting by penalizing the coefficients of less significant predictors. The degree of regularization is controlled by adjusting the alpha value. Subsequently, a random forest classifier was applied to enhance model accuracy and robustness by integrating predictions from multiple decision trees constructed on randomly selected subsets of the data. To optimize the utility of our limited training dataset (t0) and enhance the robustness of our model, leave-one-out cross-validation (LOOCV) was employed during the training phase. The classifiers’ performance was further evaluated on the t2 dataset using the receiver operating characteristic (ROC) curve and the area under the curve (AUC). Survival curves for the predicted groups were generated using the Kaplan–Meier method and compared using the log-rank test. All analyses were conducted using Python or GraphPad Prism version 9. A *p*-value < 0.05 was considered significant.

## Results

### Prospective patient collection and grouping

The duration of ECMO support is associated with mortality [18]. Accordingly, we prospectively enrolled 34 patients, classifying them into success and failure groups based on their early survival and outcomes within 7 days. The same 34 patients were also categorized as survivors or non-survivors based on the in-hospital mortality. The baseline clinical characteristics, demographic statistics, preexisting ailments, and pre- and post-ECMO variables for the success/failure groups are detailed in Supplementary Table 1, while those for the survivor/non-survivor groups are provided in Table 1.

**Table 1:** Comparison of demographic and clinical characteristics of cardiogenic extracorporeal membrane oxygenation patients grouping by survivor and non-survivor

Variable^a^	All	Survivor	Non-survivor	*p*-value
	N = 34	N = 12	N = 22	
*Baseline variables*				
Age*	52.4 (45.7, 59.3)	52.0 (47.6, 56.7)	52.4 (42.4, 59.2)	0.880
*Gender*				0.714
Male	24 (71%)	8 (67%)	16 (73%)	
Female	10 (29%)	4 (33%)	6 (27%)	
BMI (kg/m^2^)*	24.0 (21.9, 28.1)	24.0 (22.7, 25.6)	24.0 (20.7, 28.8)	0.989
*Preexisting comorbidity*				
Coronary heart disease	19 (56%)	7 (58%)	12 (55%)	1.000
Diabetes mellitus	26 (77%)	12 (100%)	14 (64%)	0.030
Dialysis	29 (85%)	12 (100%)	17 (77%)	0.137
Hypertension	19 (56%)	8 (67%)	11 (50%)	0.476
Smoking	28 (82%)	10 (83%)	18 (82%)	1.000
*NYHA scale*				0.263
I	18 (53%)	9 (75%)	9 (41%)	
II	3 (9%)	1 (8%)	2 (9%)	
III	8 (24%)	1 (8%)	7 (32%)	
IV	5 (29%)	1 (8%)	4 (18%)	
*Primary diagnosis*				0.379
Acute myocardial infarction	15 (44%)	5 (42%)	10 (45%)	
Dilated cardiomyopathy	9 (26%)	2 (17%)	7 (32%)	
Acute myocarditis	6 (18%)	4 (33%)	2 (9%)	
Arrhythmia	2 (6%)	1 (8%)	1 (5%)	
Dissection of aortic aneurysm	2 (6%)	0 (0%)	2 (9%)	
*Pre and during ECMO parameters*				
Glasgow coma scale				1.000
Severe, 3–8	12 (35%)	4 (33%)	8 (36%)	
Moderate, 9–12	4 (12%)	1 (8%)	3 (14%)	
Minor, 13–15	18 (53%)	7 (58%)	11 (50%)	
Bicarbonate infusion	19 (56%)	8 (67%)	11 (50%)	0.476
Action dialysis	13 (38%)	6 (50%)	7 (32%)	0.462
Action reperfusion	14 (41%)	3 (25%)	11 (50%)	0.275
BSA dose (m^2^)*	1.7 (1.5, 1.9)	1.7 (1.6, 1.8)	1.7 (1.5, 1.9)	0.645
Total bilirubin (µmol/L)*	36.4 (35.5, 36.9)	36.4 (35.7, 36.9)	36.5 (35.5, 36.9)	0.524
Heart Rate (beats/min)*	120 (89, 135)	124 (115, 143)	110 (87, 126)	0.062
Respiratory Rate (breath/min)*	16 (12, 20)	18 (16, 22)	14 (12, 18)	0.176
Pre-ECMO blood pressure*				
SBP (mm Hg)	91 (82, 106)	102 (93, 122)	88 (78, 95)	0.065
DBP (mm Hg)	55 (45, 65)	65 (50, 68)	53 (45, 58)	0.518
CVP (mm Hg)	15 (12, 18)	12 (12, 15)	15 (13, 18)	0.290
Pre-ECMO ventilator settings*				
PaO2/FiO2	110 (57, 270)	96 (60, 161)	128 (57, 282)	0.318
FiO2 (%) ^a^	82.1 (24.2)	83.8 (23.5)	81.1 (25.2)	0.763
Pre-ECMO blood gas*				
pH	7.3 (7.2, 7.5)	7.4 (7.2, 7.4)	7.3 (7.2, 7.5)	0.859
PaCO2 (mmHg)	33.0 (27.5, 43.7)	35.0 (31.4, 47.0)	31.7 (27.4, 43.7)	0.553
PaO2 (mmHg)	88 (57, 157)	81 (60, 91)	106 (57, 177)	0.505
HCO3 (mmol/L)	18.9 (13.7, 24.0)	20.4 (13.8, 24.4)	18.0 (13.0, 21.4)	0.362
Base excess (mmol/L)	− 4.8 (− 13.7, − 0.6))	− 5.4 (− 10.4, − 2.3)	− 4.1 (− 13.7, − 0.6)	0.901
Na (mEq/L)	138 (133, 142)	136 (134, 141)	140 (133, 143)	0.242
K (mEq/L)	4.2 (3.9, 4.9)	4.9 (4.0, 5.2)	4.1 (3.9, 4.5)	0.139
Lactate (mmol/L)	7.5 (3.1, 12.1)	7.4 (4.9, 8.8)	7.5 (2.9, 14.4)	0.530
*ECMO support parameters*				
Pre-ECMO CPR	23 (68%)	9 (75%)	14 (64%)	0.705
During ECMO CPR	22 (65%)	9 (75%)	13 (59%)	0.465
Duration of ECMO (h)*	79.1 (55.6, 160.1)	143.7 (86.4, 157.3)	68.9 (39.2, 160.0)	0.682

Data are given as n (%) or median, interquartile range (IQR)

BMI, Body mass index; BSA, body surface area; CPR, cardiopulmonary resuscitation; CVP, central venous pressure; DBP, diastolic blood pressure; ECMO, extracorporeal membrane oxygenation; FiO2, fraction of inspired oxygen; NYHA, New York Heart Association heart failure classification system; PaCO2, partial pressure of arterial carbon dioxide; PaO2, partial pressure of arterial oxygen; SBP, systolic blood pressure

^*^Welch two sample *t* test analysis; otherwise, using Fisher exact test analysis

^a^Mean ± standard deviation (± SD)

### Clinical assessments in predicting outcomes within 7 days and in-hospital mortality

**Fig. 1:**
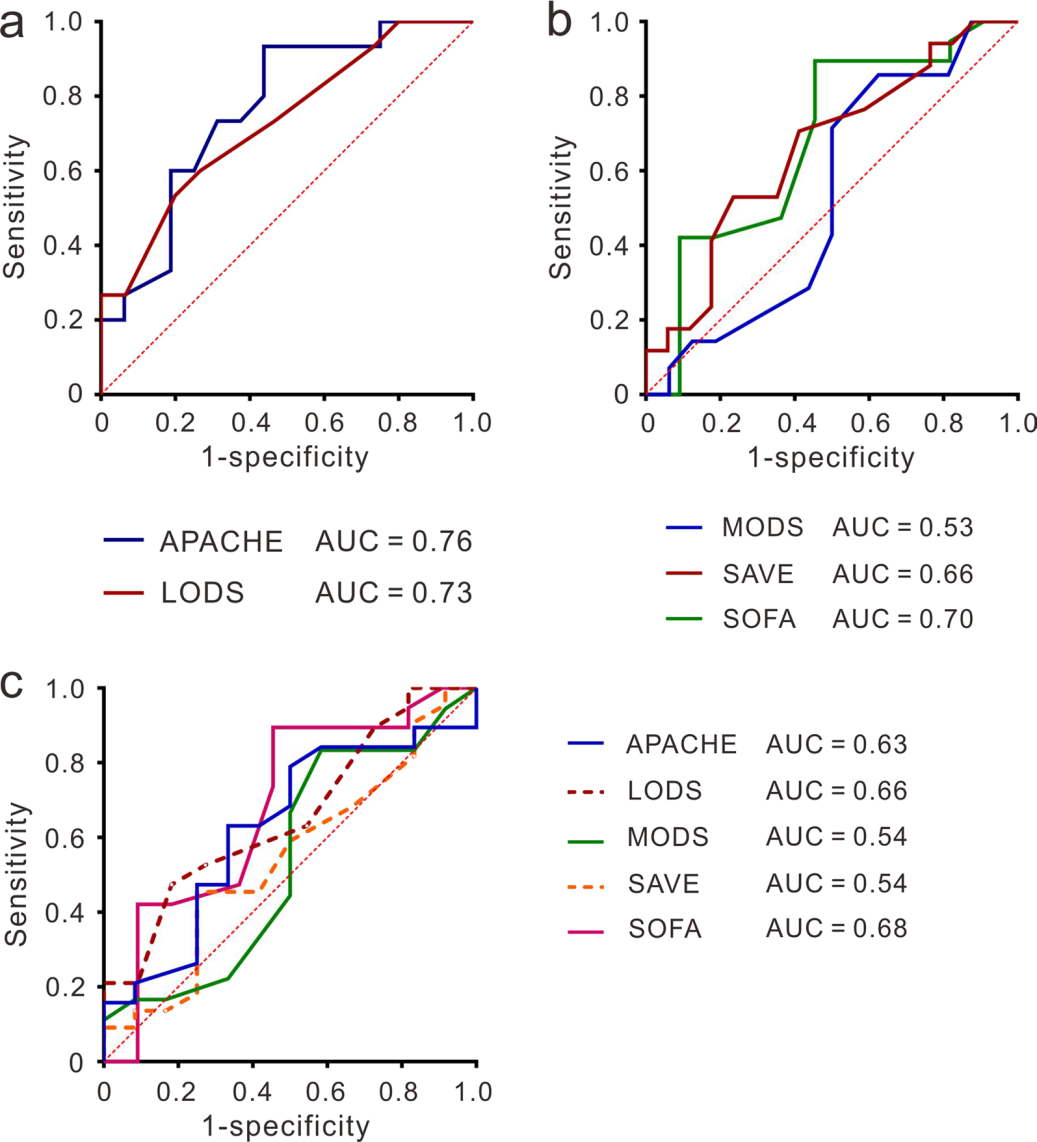
Clinical scores in predicting outcomes for CS patients receiving ECMO treatment. **a** The predictive performance of APACHE II and LODS for the outcomes within 7 days. All *p*-values for AUC are less than 0.05. **b** The predictive performance of MODS, SAVE and SOFA for the outcomes within 7 days. All *p*-values for AUC are greater than 0.05. **c** The predictive performance of APACHE II, LODS, MODS, SAVE and SOFA for in-hospital mortality. All *p*-values for AUC are greater than 0.05

Various risk assessments, including acute physiology and chronic health evaluation II (APACHE II), logistic organ dysfunction score (LODS), multiple organs dysfunction score (MODS), and sequential organ failure assessment scores (SOFA) and SAVE, were utilized to predict outcomes within our cohort. For outcomes within 7 days, both APACHE II and LODS demonstrated significant predictive power in distinguishing between success and failure groups, achieving area under the curve (AUC) values of 0.76 and 0.73, respectively (*p* < 0.05) (Fig. 1a). However, MODS, SAVE, and SOFA scores showed poor predictive performance (*p* > 0.05) (Fig. 1b). Additionally, none of these scores significantly predicted in-hospital mortality (Fig. 1c).

### Filtering the epigenetic features for model development

**Fig. 2:**
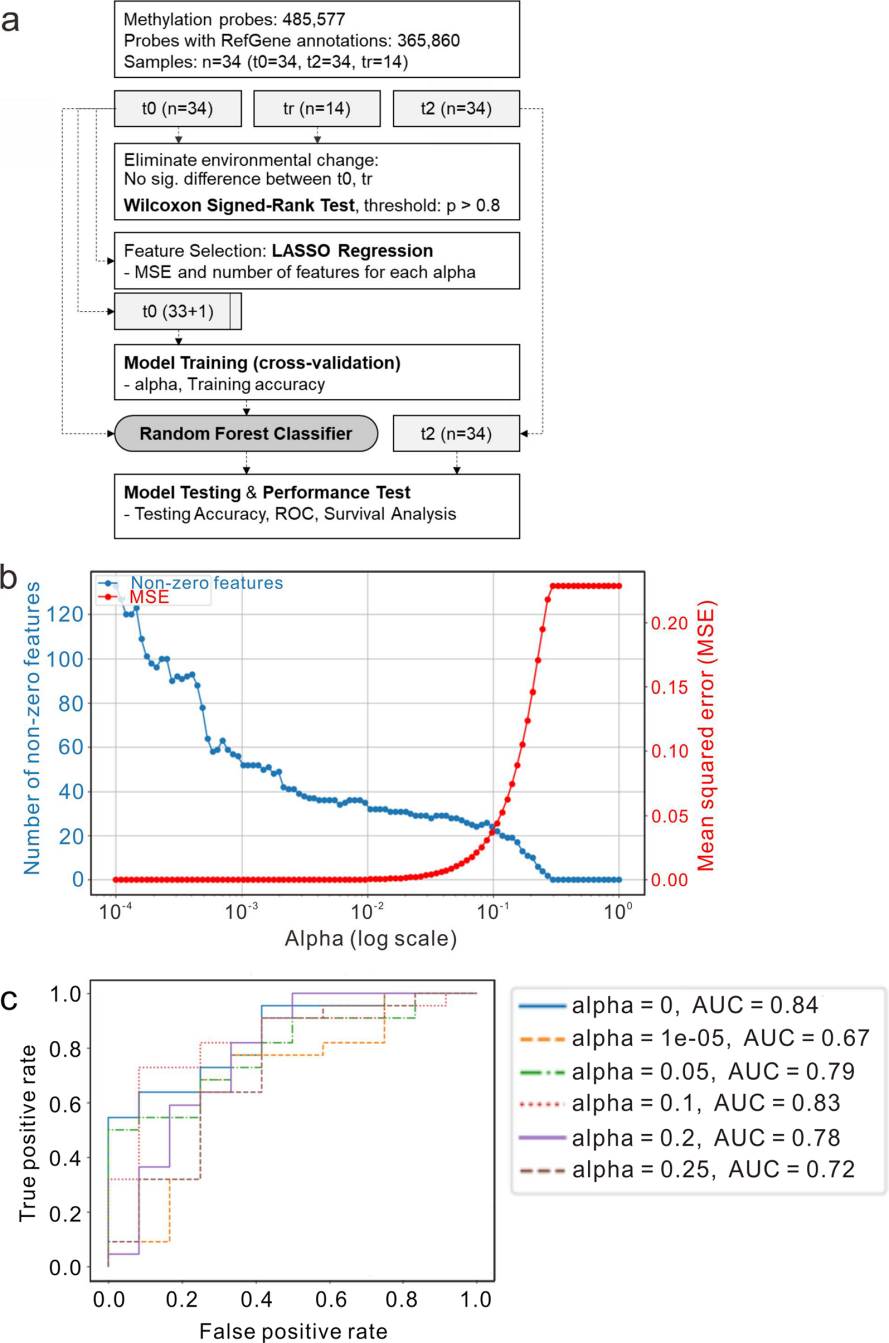
Model for the prediction of hospital mortality in CS patients with ECMO installation. **a** The workflow shows the procedure of model training and testing. **b** Results of different alphas using LASSO for feature selection. **c** ROC curves of model training performance for each tested alpha in testing cohort (t2)

To develop a biomarker capable of predicting the hospital mortality for CS patients receiving ECMO support, we profiled the epigenomics of PBMCs collected at three key stages: at the initiation of ECMO (t0), two-hour post-initiation (t2), and upon successful ECMO removal (tr). Samples were collected from 34 patients at 0 and 2 h after ECMO installation, and from 14 patients who successfully concluded ECMO support. The datasets for these different time points were denoted as t0, t2, and tr, respectively. Figure 2a illustrates the data processing workflow.

A total of 485,577 methylation probes for the Human Methylation 450 Beadchip assay were initially screened according to the annotation file. Only probes with UCSC RefGene annotations were retained, indicating their association with genes, leaving 365,860 probes for further analysis. We hypothesized that changes in methylation levels among different time points could be attributed to environmental changes resulting from ECMO treatment. For instance, methylation changes following ECMO support could reflect the effects of ECMO itself. Therefore, features that consistently represent the epigenetic characteristics of a patient across these transitions, rather than those that change due to ECMO, are considered candidates for predicting outcomes of ECMO support.

To mitigate the influence of environmental changes, we employed the Wilcoxon sign-rank test to evaluate the methylation levels between datasets t0 and tr. Probes yielding a *p*-value greater than 0.8 were deemed to exhibit no significant changes across these time points. Consequently, a total of 40,112 probes were identified and retained as stable probes.

### Developing a machine learning model to predict survival outcomes in ECMO-supported patients using temporal data sets

To develop a machine learning model capable of distinguish between survival and non-survival groups among ECMO-supported patients, we utilized t0 dataset as the training dataset. This dataset was employed to train the classification model. Subsequently, t2 dataset was used as the test dataset to evaluate the model’s classification performance. This approach ensures that our model is tested on data representing a different time point, which helps in assessing its predictive accuracy and robustness. Moreover, our methodology was tailored to overcome the constraints of a small sample size. We employed LASSO regression to minimize overfitting and enhance model validity, and utilized LOOCV to ensure robustness despite limited data. This approach significantly improved the reliability of our findings.

#### Feature selection using LASSO regression

LASSO regression was initially applied to the 40,112 stable probes in t0 dataset for feature selection. Figure 2b illustrates the mean squared error and the number of nonzero features associated with each alpha value, aiding in the determination of the optimal LASSO shrinkage parameter, alpha. Subsequently, selected alpha values of 0, 0.00001, 0.05, 0.1, 0.2, and 0.25, were tested to evaluate the classification performance. An alpha of 0 implies that all stable probes were included in the model without feature selection, using a random forest classification approach. The number of features retained for each tested alpha were 40,112; 290; 27; 24; 10; and 4, respectively.

Table 2 lists selected features for tested alpha 0.05, 0.1, 0.2, and 0.25, detailing the corresponding LASSO coefficients and Mann–Whitney *U* test *p*-values. Notably, four probes, cg25974901, cg26330738, cg04937029, and cg07137336, were selected when alpha was set to 0.25 for LASSO regression. These probes correspond to the genes PLA2G5, PTK2B, ANKRD11, and COLEC12, respectively. The p-values from the Mann–Whitney *U* test for these probes are 0.002, 0.001, < 0.001, and 0.001, respectively, indicating significant differences between the survival and non-survival groups. Noteworthy, probes cg25974901 (PLA2G5) and cg26330738 (PTK2B) are consistently selected across all four tested alpha levels, underscoring their potential significance in the dataset.

**Table 2:** Selected feature probes using LASSO regression

	Alpha-LASSO coefficient (*p*-value)					
ProbeID	0.05	0.1	0.2	0.25	InList	UCSC RefGene
cg25974901	0.049 (0.002)	0.050 (0.002)	0.036 (0.002)	0.012 (0.002)	4	PLA2G5
cg26330738	0.102 (0.001)	0.088 (0.001)	0.049 (0.001)	0.025 (0.001)	4	PTK2B
cg00207865	− 0.024 (0.001)	− 0.018 (0.001)	− 0.004 (0.001)	–	3	KCNA2
cg01302066	0.033 (0.003)	0.023 (0.003)	0.005 (0.003)	–	3	SPATA2
cg04056179	− 0.066 (0.001)	− 0.053 (0.001)	− 0.025 (0.001)	–	3	MOBKL3
cg04937029	–	0.019 (< 0.001)	0.032 (< 0.001)	0.022 (< 0.001)	3	ANKRD11
cg06612122	0.042 (0.002)	0.046 (0.002)	0.015 (0.002)	–	3	LOC644649
cg07137336	–	− 0.007 (0.001)	− 0.013 (0.001)	< − 0.001 (0.001)	3	COLEC12
cg18459475	0.051 (0.001)	0.042 (0.001)	0.005 (0.001)	–	3	AFAP1
cg27128734	− 0.046 (0.002)	− 0.029 (0.002)	− 0.005 (0.002)	–	3	KLK4
cg01050704	− 0.034 (0.012)	− 0.020 (0.012)	–	–	2	MZF1;LOC100131691
cg03955175	− 0.023 (0.014)	− 0.003 (0.014)	–	–	2	C7orf68
cg05093113	0.016 (0.003)	0.012 (0.003)	–	–	2	MIR645;PTPN1
cg06221172	0.008 (0.002)	0.003 (0.002)	–	–	2	RABL2A
cg08834922	0.021 (0.017)	0.013 (0.017)	–	–	2	AOC3
cg10436026	− 0.022 (0.002)	− 0.006 (0.002)	–	–	2	SMAD9
cg10625504	0.042 (0.008)	0.021 (0.008)	–	–	2	DHX30
cg14313868	− 0.025 (0.010)	− 0.015 (0.010)	–	–	2	NDC80;METTL4
cg17766560	0.014 (0.001)	0.009 (0.001)	–	–	2	MYO1F
cg20014942	0.008 (0.005)	0.012 (0.005)	–	–	2	FAM19A5
cg20162599	0.003 (0.005)	0.003 (0.005)	–	–	2	FERMT3
cg21863721	0.008 (0.004)	0.013 (0.004)	–	–	2	C6orf94;LTV1
cg24413879	− 0.034 (0.002)	− 0.022 (0.002)	–	–	2	HMGXB4
cg00308984	–	< − 0.001 (0.006)	–	–	1	NHEDC1
cg01062113	− 0.005 (0.004)	–	–	–	1	PANX1
cg05155965	− 0.013 (0.008)	–	–	–	1	NR2F2
cg10217166	− 0.008 (0.002)	–	–	–	1	PPM1H
cg13090402	0.007 (0.007)	–	–	–	1	TTC15
cg16524488	0.009 (0.008)	–	–	–	1	SLC12A7
cg16792234	− 0.002 (0.029)	–	–	–	1	SLC25A13

#### Evaluation of random forest classifier performance on t0 and t2 datasets

To assess the effectiveness of random forest classifiers, we trained the models using t0 dataset with different feature sets identified by LASSO regression. The performance of these models was subsequently tested using t2 dataset. Table 3 presents the LOOCV training results using t0 dataset and the testing performance on t2 dataset. The LOOCV accuracy for alphas 0.1 and 0.2 reached approximately 0.912, indicating robust training performance. Notably, alpha 0.25 demonstrated the highest testing accuracy at 0.765. Figure 2c shows ROC curves, detailing the testing performance for each alpha, highlighting the predictive capabilities of the models at different levels of feature selection.

**Table 3:** Classification performance

Performance	LASSO alpha					
	0	0.00001	0.05	0.1	0.2	0.25
# of Features	40,112	290	27	24	10	4
*Random forest classifier (T0 dataset, leave-one-out cross-validation)*						
LOOCV accuracy	0.559 ± 0.497	0.618 ± 0.486	0.853 ± 0.354	0.912 ± 0.284	0.912 ± 0.284	0.794 ± 0.404
LOOCV precision	0.559 ± 0.497	0.618 ± 0.486	0.647 ± 0.478	0.647 ± 0.478	0.647 ± 0.478	0.618 ± 0.486
LOOCV recall	0.559 ± 0.497	0.618 ± 0.486	0.647 ± 0.478	0.647 ± 0.478	0.647 ± 0.478	0.618 ± 0.486
LOOCV F1 score	0.559 ± 0.497	0.618 ± 0.486	0.647 ± 0.478	0.647 ± 0.478	0.647 ± 0.478	0.618 ± 0.486
*Random forest classifier (T0 training, self-test)*						
Training accuracy	1	1	1	1	1	1
Training precision	1	1	1	1	1	1
Training recall	1	1	1	1	1	1
Training F1 score	1	1	1	1	1	1
*Random forest classifier (T0 training, T2 testing)*						
Test accuracy	0.647	0.647	0.706	0.706	0.735	0.765
Test precision	0.647	0.647	0.688	0.700	0.710	0.769
Test recall	1	1	1	0.955	1	0.909
Test F1 score	0.786	0.786	0.815	0.808	0.83	0.833

**Fig. 3:**
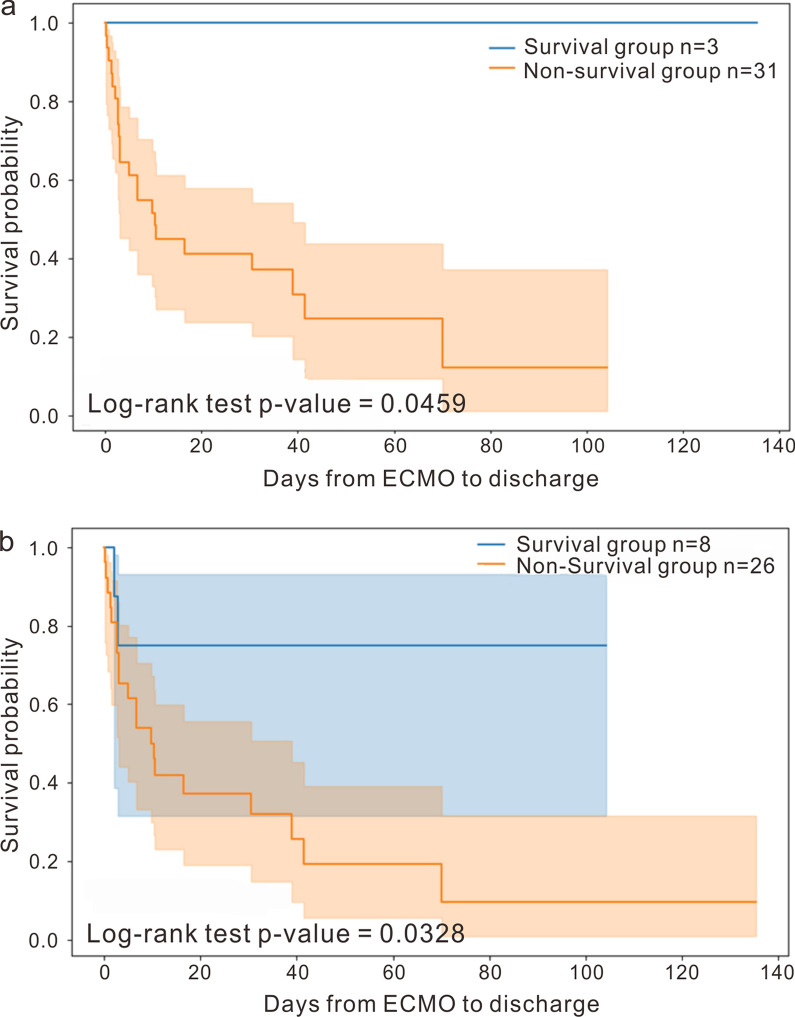
Kaplan–Meier survival analysis for ten and four-feature models in testing dataset. **a** In-hospital survival is analyzed by the ten-feature classification, where features are selected based on an alpha setting of 0.20 for LASSO regression. **b** In-hospital survival is analyzed by the four-feature classification, where features are selected based on an alpha setting of 0.25 for LASSO regression. Significance is determined by the log-rank test (*p* < 0.05)

Given that ten features from LASSO alpha 0.20 and four features from alpha 0.25 demonstrated better predictive accuracy on the t2 testing dataset (0.735 and 0.765, respectively), we sought to determine their association with in-hospital survival. Consequently, we conducted Kaplan–Meier survival analyses using these feature sets. The results from the classifiers at LASSO alphas of 0.20 and 0.25 showed significant differences. The ten-feature classifier had a *p*-value of 0.0459 (Fig. 3a), while the four-feature classifier had a *p*-value of 0.0328 (Fig. 3b), indicating significant differences in survival between patients predicted to be in the survival or non-survival groups, as determined by the log-rank test. Collectively, the methylated ten-feature and four-feature sets proved to be superior biomarkers for predicting outcomes in CS patients undergoing ECMO treatment.

#### The development of EpiSAVE classifier

The clinical characteristics contribute predictive power to current scoring systems, including the SAVE score, whose importance and significance are well-recognized in the clinical setting. Therefore, we incorporated the SAVE score with selected methylation features and retrained the classifier models, naming them 'EpiSAVE.' The final results show enhanced AUC values for the ROC curves, improving to 0.82 from 0.78 at an alpha value of 0.2, and to 0.74 from 0.72 at an alpha value of 0.25 in the t2 database (Supplementary Fig. 1). Survival curves show significant differences between two groups when using the EpiSAVE classifiers at alpha values of 0.2 and 0.25 (log-rank test, *p* < 0.05) (Supplementary Fig. 2a and b). Collectively, the methylation ten-feature and four-feature sets combined with SAVE score show potential as prognostic biomarkers for CS patients undergoing ECMO treatment in the real clinical setting.

## Discussion

Despite the improvement in patient outcomes with ECMO compared to conventional CPR, in-hospital survival rates remain below 50% [8, 20]. Research into prognostic markers at the molecular level is limited, yet it is crucial for efficiently managing patients receiving ECMO and for optimizing the use of medical resources. To address the deficiency of reliable prognostic markers, this study employed machine learning models to develop DNA methylation features in PBMC profiles and these models were tested using the dataset at time point t2. We identified two classifiers of methylation features as independent prognostic markers for CS patients undergoing ECMO treatment.

Appropriate patient identification for ECMO use presents significant challenges, as current guidelines from the Extracorporeal Life Support Organization (ELSO) for managing cardiogenic shock (CS) do not incorporate prediction tools [21, 22]. Given the complexity of the disease before ECMO initiation and the high risk of ECMO-related complications, the survival rate until hospital discharge is approximately 30–45% [6, 8, 23]. The development of a prognostic marker to identify critically ill patients who could benefit from ECMO is an unmet medical need. The first reported model for predicting survival in adults with refractory cardiogenic shock (CS) supported by ECMO was the Survival After Venoarterial-ECMO (SAVE) score, introduced in 2015 [6]. The parameters of the SAVE score include age, weight, blood pressure, and pre-ECMO organ failures, among others. However, a recent study indicated that its calibration is limited, as it tends to underestimate survival rates in adult patients with cardiogenic shock [22]. Several pre-ECMO risk factors have been identified, including diabetes, obesity, renal insufficiency, and lactate levels [6, 24–26]. In line with these risk factors, our study reveals that failure group have lower heat rates and lower blood bicarbonate levels. However, an unexpected correlation between outcomes and diabetes was observed (Table 1 and Supplementary Table 1), which could likely be attributed to the small sample size. In advanced heart failure patients undergoing mechanical circulatory support, the transcriptome profiles of preoperative PBMCs predict early improvement of organ function with an average prediction accuracy of 93% and correlate with one-year survival [13]. The study identified several immune-related candidates as the prognostic biomarkers. Furthermore, Yang et al. found that successful ECMO treatment in acute myocardial infarction (AMI) combined with CS patients exhibited a lower proportion of natural killer T cells and identified two genes, ASB13 and CDCA7, associated with the progression [27]. In our previous study, cytokine IL-10 was identified as promising biomarker at t0, whereas the level of IL-10 exhibited variations across different time points [11, 12]. In contrast, our epigenetic features demonstrated consistency at time points t0, t2, and tr. Notably, IL-10 levels above 16.58 pg/ml differentiated survivors from non-survivors among AMI patients, and levels above 143.17 pg/ml were indicative for DCMP patients, demonstrating the underlying diseases of CS are associated with cytokines. In particular, elevated procalcitonin and lower VEGF in the blood on the second day, while supported by a VA-ECMO device, are significantly associated with an increased mortality risk [28, 29]. These findings suggest that protein levels in blood vary over time and contribute to the causes of cardiogenic shock. Therefore, the DNA methylation–derived epigenetic scores for circulating protein levels were development and that associated with incident CVD and could be a significant predictor of CVD risk, independent of the concentration of troponin I (cTnI), which is an important cardiac injury marker [30, 31]. Epigenetic feature is such a powerful marker that shows potential for broader application across various conditions, and even can serve as a predictor for all-cause mortality. This is because it encapsulates both genetic predispositions and environmental influences [32, 33]. Therefore, utilizing t2 as the testing dataset to verify the predictive efficacy of the DNA methylation features not only underscores the robustness of methylation features, but also suggests that the substantial utility of these features may remain consistent across different time frames of ECMO treatment.

Methylation on the genes Protein Tyrosine Kinase 2 Beta (PTK2B) and Phospholipase A2, Group V (PLA2G5), are significant features in the predictive models across all tested alphas, indicating their substantial roles. Pyk2, a Ca2+ -dependent non-receptor tyrosine kinase, transduces signals from Ca2+ to the downstream mitogen-activated protein kinase signaling pathway [34]. PTK2B is activated in nonischemic failing human ventricles and is involved in cardiac arrhythmogenesis through the regulation of gene expression related to Ca2+ flux in the Pyk2−/− mouse model [35]. Inhibiting Pyk2 activity has been shown to reduce infarct size and improve cardiac function in myocardial infarction heart failure rats [36]. PLA2G5, a member of the superfamily of PLA2 enzymes, is characterized by its ability to hydrolyze the center (sn-2) ester bond of phospholipids, generating free fatty acid and lysophospholipid [37]. LDL receptor-deficient mice, either overexpressing or deficient in PLA2G5, provide in vivo evidence that Group V secretory phospholipase A2 (sPLA2-V) contributes to atherosclerosis [38]. Furthermore, specific PLA2G5 tagging single-nucleotide polymorphism (tagSNP) haplotypes have shown a significant association with plasma low-density lipoprotein (LDL), total cholesterol, and oxidized LDL/LDL levels [39]. Collectively, these evidences suggest that the methylated PTK2B and PLA2G5 in PBMCs may play critical roles in mediating heart failure and outcomes beyond simply serving as markers.

To evaluate the comparability of methylation predictive models, several commonly used scoring systems in the Intensive Care Unit (ICU) such as APACHE II [40], LODS [41], MODS [42], and SOFA [43] and SAVE were analyzed. However, none of these scores could predict the final in-hospital mortality. The prognostic value of the APACHE II score has been validated in CS patients treated with a percutaneous left ventricular assist device, achieving an AUC of 0.70 with a 95% CI of 0.62–0.78 [44]. The LODS score was developed by analyzing data from 14,745 ICU admissions in 12 different countries in 1996 [41] and further validated in cardiac surgical patients in 2011 [45]. In our cohort, both the APACHE II and LODS scores demonstrated significant predictive power for 7-day outcomes (success/failure), as shown in Fig. 1a. Both ICU scoring systems, which rely solely on clinical characteristics, effectively predict early-stage mortality in CS patients receiving ECMO support. This suggests that clinical characteristics are primarily indicators of short-term outcomes. In contrast, molecular features in PBMCs, particularly DNA methylation patterns, likely reflect the underlying conditions that determine long-term prognosis.

### Study limitations

First, this study involves prospective single-center enrollment and analysis, which raises concerns about biased selection and unmeasured confounding factors. Second, the limited sample size of only 34 CS patients, due to difficulties in recruiting ECMO-supported CS patients and the absence of external validation cohorts pose challenges. Notably, diabetes patients exhibited a positive correlation with survivor/success group, a finding that is typically less common in larger or retrospective cohorts. Third, the high-dimensional features (485,577 probes) combined with a small sample size can easily lead to model overfitting. To address these challenges, we applied LASSO, a random forest classifier and LOOCV. LASSO helps in reducing the number of features to a manageable size, while the random forest classifier, by building multiple decision trees on random subsets of the data and aggregating their results, provides a robust model that improves accuracy and mitigates the risk of overfitting. LOOCV was employed to maximize the data available for model training, thus enhancing the robustness of our findings. These methods were implemented to improve the generalizability of using epigenetic markers in our study. To the best of our knowledge, no public epigenomic databases are available for validation. However, the absence of comparable and longitudinal databases enhances the novelty and uniqueness of our study, positioning it as a pioneering approach in predicting and interpreting the outcomes of CS patients receiving ECMO.

## Conclusion

The clinical significance of this study lies in the current lack of sufficient tools to predict the outcomes of patients undergoing ECMO treatment for cardiogenic shock within the early phase of ECMO installation (within one day). Notably, the APACHE II, SAPS III [46], and SOFA scores, which were developed from databases of general internal medicine intensive care patients, are not applicable to ECMO patients. The ENCOURAGE score is specifically designed to predict 30-day mortality for acute myocardial infarction patients treated with ECMO [10, 47]. The SAVE-VA score, utilizing simple parameters, estimates the success rate of ECMO treatment. Bothe scores are intended for use before ECMO installation, not for predicting the outcomes of specific individuals after installation.

In this study, we utilize epigenomic biomarkers in conjunction with a machine learning model to predict the outcome of specific patients with cardiogenic shock treated with VA-ECMO during an early phase. The methylation features demonstrate significant predictive power for in-hospital mortality, showing superior performance compared to current clinical scores. By analyzing blood samples taken at the time of device installation and two-hour post-installation, we can differentiate patients with high versus low probabilities of survival, allowing clinicians to more aggressively search for causes and intervene timely in patients with lower survival probabilities, thereby increasing the probabilities of survival with ECMO treatment in clinical settings.

## Supplementary information

Supplementary Material 1

## Data Availability

Data supporting the findings of this study have been deposited in the Gene Expression Omnibus (GEO) at the National Center for Biotechnology Information (NCBI). The GEO accession number is GSE182609.

## Abbreviations

AMI: Acute myocardial infarction
APACHE II: Acute physiology and chronic health evaluation II
AUC: Area under the curve
CPR: Cardiopulmonary resuscitation
CS: Cardiogenic shock
DCMP: Dilated cardiomyopathy
ECMO: Extracorporeal membrane oxygenation
LASSO: Least absolute shrinkage and selection operator
LODS: Logistic organ dysfunction score
LOOCV: Leave-one-out cross-validation
MODS: Multiple organs dysfunction score
MOF: Multiple organ failure
PBMCs: Peripheral blood mononuclear cells
ROC: Receiver operating characteristic
SAVE: Survival after venoarterial ECMO
SOFA: Sequential organ failure assessment scores
