# Supplementary material for: Epigenomic biomarkers insights in PBMCs for prognostic assessment of ECMO-treated cardiogenic shock patients
Source: Clin Epigenetics. 2024 Oct 3;16:137. doi: 10.1186/s13148-024-01751-6 (PMC11451087; doi:10.1186/s13148-024-01751-6)
Supplement: Supplementary file 1 — Supplementary Material 1 [file 13148_2024_1751_MOESM1_ESM.docx]

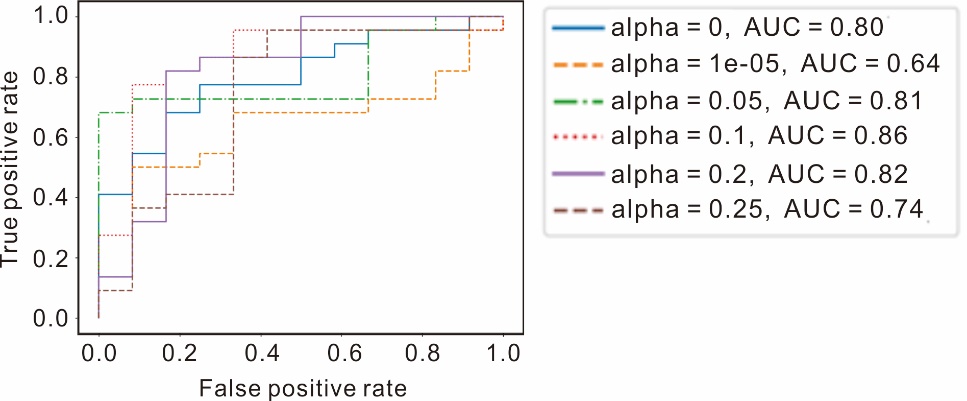


**Supplemental Figure 1.** **ROC curve performance of EpiSAVE classifiers across tested alpha values in t2 testing cohort**

Epigenetic features were initially filtered using the Wilcoxon Signed-Rank Test with a threshold of p > 0.8 between t0 and tr datasets, resulting in 40,112 probes remaining for LASSO selection. Subsequently, using the alpha value for penalization in LASSO to identify epigenetic features, and including the SAVE score as one of those features, a random forest classifier was re-trained on t0 datasets, which was then validated on t2 datasets. ROC curves for different alpha values were calculated along with their respective AUC values.


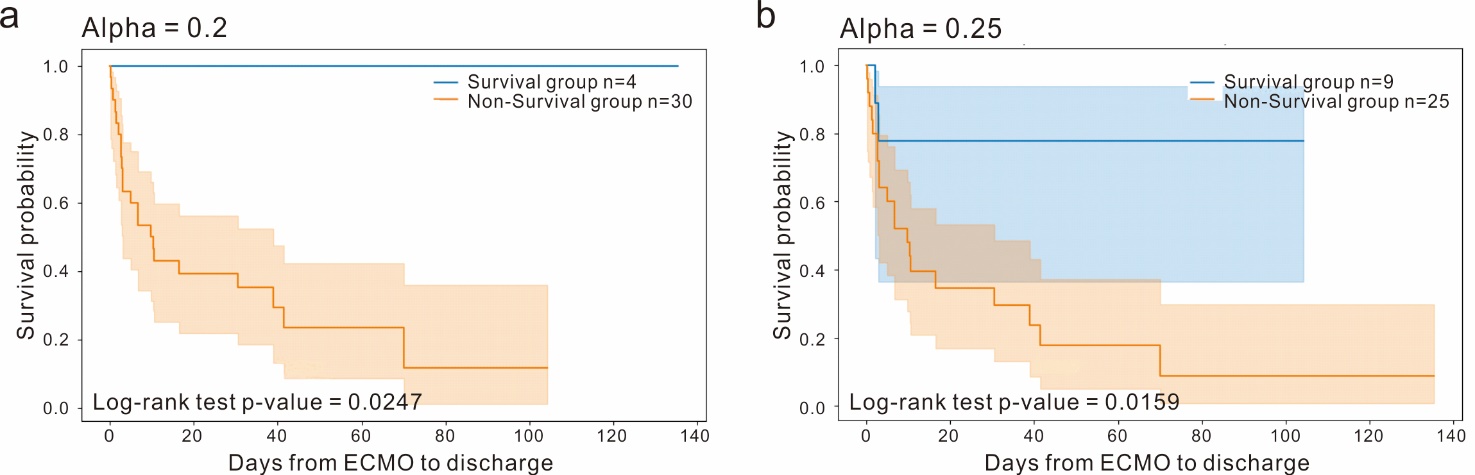


**Supplemental Figure 2. In-hospital survival based on EpiSAVE classifier using different alpha values in t2 testing cohort.**

Epigenetic features were initially filtered using the Wilcoxon Signed-Rank Test with a threshold of p > 0.8 between the t0 and tr datasets, resulting in 40,112 probes remaining for LASSO selection. Subsequently, the selected features, with an alpha value applied for penalization in LASSO, were used alongside a trained random forest classifier for validation on the t2 datasets. The Kaplan-Meier plots for (a) alpha is 0.2; (b) alpha is 0.25. The log-rank test was used to determine significance between two groups. A p-value of less than 0.05 was considered statistically significant.

**Supplemental Table 1. Comparison of demographic and clinical characteristics of cardiogenic extracorporeal membrane oxygenation patients in success and failure groups.**

| **Variable** | **All** | **Success** | **Failure** | | **p-value** |
| --- | --- | --- | --- | --- | --- |
|  | N = 34 | N = 17 | N = 17 | |  |
| Age* | 52.4 (45.7, 59.3) | 51.2 (47.0, 56.5) | 52.4 (45.2, 63) | | 0.590 |
| Gender |  |  |  | | 0.708 |
| Male | 24 (71%) | 11 (65%) | 13 (76%) | |  |
| Female | 10 (29%) | 6 (35%) | 4 (24%) | |  |
| BMI (kg/m^2^)* | 24.0 (21.9, 28.1) | 23.0 (21.8, 25.2) | 25.0 (22.4, 28.9) | | 0.329 |
| **Pre-existing comorbidity** |  |  |  | |  |
| Coronary heart disease | 15 (44%) | 7 (41%) | 8 (47%) | | 1.000 |
| Diabetes mellitus | 26 (77%) | 16 (94%) | 10 (59%) | | 0.039 |
| Dialysis | 5 (29%) | 2 (12%) | 3 (18%) | | 1.000 |
| Hypertension | 15 (44%) | 6 (35%) | 9 (53%) | | 0.491 |
| Smoking | 6 (18%) | 3 (18%) | 3 (18%) | | 1.000 |
| NYHA scale |  |  |  | | 0.764 |
| I | 18 (53%) | 10 (59%) | 8 (47%) | |  |
| II | 3 (9%) | 2 (12%) | 1 (6%) | |  |
| III | 8 (24%) | 3 (18%) | 5 (29%) | |  |
| IV | 5 (29%) | 2 (12%) | 3 (18%) | |  |
| **Diagnosis Group** |  |  |  | | 0.415 |
| Acute myocardial infarction | 15 (44%) | 7 (41%) | 8 (47%) | |  |
| Dilated cardiomyopathy | 9 (26%) | 4 (24%) | 5 (29%) | |  |
| Acute myocarditis | 6 (18%) | 4 (24%) | 2 (12%) | |  |
| Arrhythmia | 2 (6%) | 2 (12%) | 0 (0%) | |  |
| Dissection of aortic aneurysm | 2 (6%) | 0 (0%) | 2 (12%) | |  |
| **Pre and during ECMO parameters** | |  |  |  |  |
| Glasgow coma scale |  |  |  | | 0.349 |
| Severe, 3-8 | 12 (35%) | 4 (24%) | 8 (47%) | |  |
| Moderate, 9-12 | 4 (12%) | 2 (12%) | 2 (12%) | |  |
| Minor, 13-15 | 18 (53%) | 11 (65%) | 7 (41%) | |  |
| Bicarbonate infusion | 15 (44%) | 5 (29%) | 10 (59%) | | 0.166 |
| Action Dialysis | 21 (62%) | 9 (53%) | 12 (71%) | | 0.481 |
| Action Reperfusion | 20 (59%) | 13 (77%) | 7 (41%) | | 0.080 |
| BSA dose (m^2^)* | 1.7 (1.5, 1.9) | 1.7 (1.5, 1.7) | 1.8 (1.6, 1.9) | | 0.172 |
| Total bilirubin (µmol/L)* | 36.4 (35.5, 36.9) | 36.5 (36.1, 36.9) | 36.0 (35.3, 36.8) | | 0.280 |
| Heart Rate (beats/min)* | 120 (89, 135) | 124 (108, 142) | 98 (84, 124) | | 0.037 |
| Respiratory Rate (breath/min)* | 16 (12, 20) | 16 (14, 21) | 15 (12, 19) | | 0.826 |
| **Pre-ECMO blood pressure*** |  |  |  | |  |
| SBP (mm Hg) | 91 (82, 106) | 94 (87, 119) | 88 (78, 97) | | 0.402 |
| DBP (mm Hg) | 55 (45, 65) | 60 (53, 65) | 51 (45, 69) | | 0.795 |
| CVP (mm Hg) | 15 (12, 18) | 12 (12, 17) | 15 (15, 19) | | 0.225 |
| **Pre-ECMO ventilator settings*** | |  |  |  |  |
| PaO_2_/FiO_2_ | 110 (57, 270) | 110 (62, 206) | 139 (57, 275) | | 0.720 |
| FiO_2_ (%) ^a^ | 82.1 (24.2) | 81.5 (23.7) | 82.7 (25.6) | | 0.888 |
| **Pre-ECMO blood gas*** |  |  |  | |  |
| pH | 7.3 (7.2, 7.5) | 7.4 (7.3, 7.5) | 7.2 (7.1, 7.4) | | 0.199 |
| PaCO_2_ (mmHg) | 33.0 (27.5, 43.7) | 33.3 (30.5, 39.7) | 31. 7 (27.4, 45.7) | | 0.961 |
| PaO_2_ (mmHg) | 88 (57, 157) | 83 (60, 105) | 106 (57, 177) | | 0.800 |
| HCO_3_ (mmol/L) | 18.9 (13.7, 24.0) | 21.1 (17.0, 24.6) | 17.6 (12.8, 19.4) | | 0.035 |
| Base excess (mmol/L) | -4.8 (-13.7, -0.6) | -3.6 (-7.2, 1.1) | -8.5 (-15.2, -2.7) | | 0.065 |
| Na (mEq/L) | 138 (133, 142) | 136 (132, 141) | 141 (137, 144) | | 0.082 |
| K (mEq/L) | 4.2 (3.9, 4.9) | 4.6 (4.0, 5.0) | 4.1 (3.9, 4.6) | | 0.315 |
| Lactate (mmol/L) | 7.5 (3.1, 12.1) | 6.8 (2.7, 8.8) | 10.0 (3.4, 14.8) | | 0.113 |
| **ECMO support parameters** | |  |  |  |  |
| Pre-ECMO CPR | 11 (32%) | 4 (24%) | 7 (41%) | | 0.465 |
| During ECMO CPR | 12 (35%) | 3 (18%) | 9 (53%) | | 0.071 |
| Duration of ECMO (hr)* | 79.1 (55.6, 160.1) | 144.4 (91.0, 218.3) | 63.3 (37.8, 119.5) | | 0.035 |

Data are given as n (%) or median, interquartile range (IQR). ^a^ mean ± standard deviation (± SD).

* Welch two sample t-test analysis; otherwise, using Fisher exact test analysis.

BMI-body mass index, BSA-body surface area, CPR-cardiopulmonary resuscitation, CVP-central venous pressure, DBP-diastolic blood pressure, ECMO-extracorporeal membrane oxygenation, FiO2-fraction of inspired oxygen, NYHA-New York Heart Association heart failure classification system, PaCO2-partial pressure of arterial carbon dioxide, PaO2-partial pressure of arterial oxygen, SBP-systolic blood pressure.
